# Using audit to enhance quality of maternity care in resource limited countries: lessons learnt from rural Tanzania

**DOI:** 10.1186/1471-2393-11-94

**Published:** 2011-11-16

**Authors:** Angelo S Nyamtema, Alise Bartsch de Jong, David P Urassa, Jos van Roosmalen

**Affiliations:** 1 Saint Francis Designated District Hospital, Ifakara, Tanzania; 2 Department of Medical Humanities, EMGO Institute for Health and Care Research, VU Medical Centre, Amsterdam, The Netherlands; 3 Department of Community Health, Muhimbili University of Health and Allied Sciences, Dar es Salaam, Tanzania; 4 Department of Obstetrics, Leiden University Medical Centre, The Netherlands

## Abstract

**Background:**

Although clinical audit is an important instrument for quality care improvement, the concept has not yet been adequately taken on board in rural settings in most resource limited countries where the problem of maternal mortality is immense. Maternal mortality and morbidity audit was established at Saint Francis Designated District Hospital (SFDDH) in rural Tanzania in order to generate information upon which to base interventions.

**Methods:**

Methods are informed by the principles of operations research. An audit system was established, all patients fulfilling the inclusion criteria for maternal mortality and severe morbidity were reviewed and selected cases were audited from October 2008 to July 2010. The causes and underlying factors were identified and strategic action plans for improvement were developed and implemented.

**Results:**

There were 6572 deliveries and 363 severe maternal morbidities of which 36 women died making institutional case fatality rate of 10%. Of all morbidities 341 (94%) had at least one area of substandard care. Patients, health workers and administration related substandard care factors were identified in 50% - 61% of women with severe morbidities. Improving responsiveness to obstetric emergencies, capacity building of the workforce for health care, referral system improvement and upgrading of health centres located in hard to reach areas to provide comprehensive emergency obstetric care (CEmOC) were proposed and implemented as a result of audit.

**Conclusions:**

Our findings indicate that audit can be implemented in rural resource limited settings and suggest that the vast majority of maternal mortalities and severe morbidities can be averted even where resources are limited if strategic interventions are implemented.

## Background

Every year worldwide there are 358,000 maternal deaths; 99% of these take place in resource limited countries and 65% of the total deaths is contributed by only eleven countries from East Asia and sub-Saharan Africa including Tanzania [1]. On the other hand, evidence indicates that most (74-98%) maternal deaths and severe disabilities can be averted even where resources are limited but, in order to do so, the right kind of information is needed upon which to base interventions [2-5]. Maternal death and severe morbidity audit is one of the ways of generating this kind of information about causes, underlying substandard care and how they can be averted [5-7]. Substandard care could be related to the patient: where a woman or her relatives caused delay that contributed to death or severe morbidity; or health care provider by delaying or mismanaging the case; or administration: where something that is the responsibility of the health authority was not available [8,9].

Although the effectiveness of audit in reducing adverse maternal outcome remains a matter of debate, it is widely practiced in the West and less frequently reported in resource limited countries particularly in rural settings [10-12]. In an attempt to improve the quality of care in rural Tanzania, an audit system for maternal mortality and severe morbidity was established at Saint Francis Designated District Hospital (SFDDH) and as a result focused interventions were developed and implemented.

## Methods

### Study area

Saint Francis Designated District Hospital is located in Kilombero, one of the rural districts in South-east Tanzania. The district has a total area of 14,018 km^2 ^and a population of 331,167 with an annual population growth rate of 2.6%. The district has only two non-governmental hospitals with comprehensive emergency obstetric care (CEmOC) services both located around 75 km apart in the northern part of the district. Because of the geographical locations of these hospitals patients requiring CEmOC services from the most southern part of the district need to travel up to 200 km to reach the closest hospital (SFDDH). SFDDH has a capacity of 372 beds and provides services to patients not only from Kilombero district but also to those from the neighboring districts, Ulanga, Kilosa and Morogoro rural. The average annual delivery rate was 4987 between 2006 and 2008.

### Study design

Methods are informed by the principles of operations research. The purpose of using principles of operations research was to apply scientific methods, techniques and tools to problems involving the operations of a health system in Tanzania so as to provide those in control of the system with optimum solutions to problems. The principles applied included (1) formulation of the problem; (2) construction of a model of the audit system; (3) selection of a solution technique; (4) obtaining a solution to the problem; (5) establishing controls over the system; and (6) implementation of the solutions [13].

### Development and validation of audit record form

The audit record form was developed and terms (severe maternal morbidities) were defined. At the time when an audit system for maternal mortality and severe morbidities was introduced in this hospital, there was no internationally accepted standard definition and uniform case-identification criteria for severe maternal morbidity [14]. Factors associated with maternal mortality and morbidities were extracted from literature. A panel of 2 experts (obstetricians) reviewed the form for relevance and clarity. Items regarded as relevant for inclusion by both experts were retained in the form. Inappropriate items were either removed or modified based on discussion. The form was piloted on 48 cases of maternal mortality and severe morbidity from May to September 2008. More revisions were made during this period based on the feedback from the team administered the audit record form and those reviewed the data collected. Data collected during this period was not included in this article. The final version of this form had several sections including: background information, socioeconomic status, antenatal care history, previous obstetric history, intrapartum care and areas of substandard care.

### Definition of terms and inclusion Criteria

Inclusion criteria were: all maternal deaths, eclampsia, severe obstructed labour (defined as those presenting with (impending) rupture of uterus, haematuria or obstetric fistula), severe obstetric haemorrhage (defined as patients who received at least one pint of blood or estimated blood loss of more than 1000 ml), severe anaemia (Hb ≤ 6 g/dl), puerperal sepsis, severe complications of abortion (defined as perforation of viscera or haemorrhage necessitating transfusion) or severe sepsis [defined as sepsis associated with organ dysfunction, hypotension, or hypoperfusion abnormalities including oliguria or alteration in mental status]), ruptured ectopic pregnancy and any other obstetric complications which the doctors were convinced to be severe maternal morbidities. All patients fulfilling the inclusion criteria were reviewed and selected cases were audited.

### Data Collection

Data collection was done in blocks for 491 days from October 6^th ^2008 until July 8^th ^2010 using a semi-structured audit record form. While the forms for maternal mortality were completed on the day of the event, those for severe morbidities were completed on the day of discharge. In both cases (mortality and morbidity) copies of the case files and partograms (whenever applicable) were attached.

All cases were discussed in the first place by the senior obstetrician, intern doctors and the medical students involved in the data collection, to establish the cause of severe morbidity or mortality and the related substandard care. The management of the case was assessed and judged against the national guidelines for management of emergency obstetric conditions. In case of missing information in case files the staff who attended the patient and whenever the patient was still in the ward were asked for clarification. All maternal mortalities and selected severe morbidities were discussed in regular audit meetings. Selection of severe morbidity cases for audit was based on the presence of gross substandard care. In these meetings the audit team critically reviewed cases, established the cause of mortality or severe morbidity, underlying substandard care and developed strategic action plans for future improvement.

In order to reach consensus the facilitator involved as many members of the audit team as possible to give their opinions and suggestions about the case. The input and ideas of all participants were gathered, synthesized and the facilitator tested the panel to see if the listed causes, areas of substandard care and interventions were acceptable to all. Although anonymity was emphasized during audit meetings, feedback was provided later by more senior staff in case of health workers related substandard care in order to improve the future management of patients.

### Audit team

The audit team was formed based on the recommendations of the national guidelines for audit team formation at the district hospital. These constituted the hospital medical director, head of department of obstetrics and gynaecology, nurse in-charge of the maternity block, district nursing officer, district reproductive and child health (RCH) coordinator, obstetricians, representatives from RCH clinic, pharmacy, laboratory and operating theatre, and other health care providers from the maternity blocks. The health care providers in this department included 2 obstetricians, 2 generalist doctors (medical doctors not yet specialized), 2 assistant medical officers, 14 midwives. The district medical officer did not take part although he was supposed to do so by the guidelines.

### Evaluation of the auditing process

In addition to the routine evaluation of the auditing process that was carried out during every audit meeting, a summary of findings was discussed to evaluate the audit process including implementation of recommendations at the end of every two months.

### Data analysis

Data was extracted from the audit record form and entered into Access database and then transferred to the SPSS software for analysis. The characteristics and substandard care of maternal mortalities and severe morbidities were analyzed and compared within the group. The principal summary measures were case fatality rates (a widely accepted indicator for quality care [15]), proportions of the causes and substandard care as well as mortality risk ratios (RR). The corresponding 95% confidence intervals (95%CI) were also calculated. Statistical significance of the results was estimated using p-value (with a significance level, 'α', of 0.05) and 95%CI. Ethical clearance for the study was obtained from SFDDH Research and Publication Committee. The permission to conduct this audit was obtained from the district and hospital management. Verbal consent was obtained from all women included in the audit process.

## Results

### Background information

During the study period the total number of institutional deliveries at SFDDH was 6572 and there were 363 severe maternal morbidities, giving an incidence of 55 per 1000 births. Of all mothers presenting with severe morbidities 265 (73%) were married or living with their partners and 252 (78%) were primary school leavers. Adolescent (under 20 years of age) pregnancy was the most common risk factor found in nearly one third (28%) of those with severe maternal morbidities (Table 1).

**Table 1:** Risk factors among women with severe maternal morbidities admitted at SFDDH, October 2008 to July 2010

Domain	SFDDH Total Deliveries n = 6572	Maternal Morbidities n = 363
Age distribution		
< 20 years	1577 (24%)	102 (28%)
20 - 35 years	4403 (67%)	231 (64%)
36 years and above	526 (8%)	30 (8%)
Missing or did not know their age	66 (1%)	0 (0%)
Distance of village of residence from SFDDH		
Within 50 km	4075 (62%)	243 (67%)
51 - 100 km	1643 (25%)	72 (20%)
101 - 150 km	592 (9%)	14 (4%)
151+ km	131 (2%)	34 (9%)
Residential village/street not recognized*	131 (2%)	0 (0%)
Parity 5 and above	523 (9%)	32 (9%)
HIV status		
HIV positive	270 (5%)	8 (2%)
Not known (not checked)	1383 (25%)	53 (15%)

*This group included women who had registered themselves that they were coming from the regions which are far away from Ifakara. Based on the local culture it was assumed that they had come back to their parents to the nearby villages to wait for delivery. Others registered streets (instead of villages) which could not be recognized during the analysis.

### Characteristics of maternal mortalities and morbidities

Of all severe morbidities 36 mothers died, making an institutional case fatality rate of 10%. Specific case fatality rate was as high as 16% found among patients with severe abruptio placentae (Table 2). The major causes of maternal deaths were eclampsia, complications of abortion, severe anaemia in pregnancy and ruptured uterus contributing to almost two thirds (64%) of all deaths. Half (3) of all deaths caused by severe abortion complications were unsafely induced. Of all deaths, 29 (81%) developed severe complications before arrival at the hospital and almost two thirds 17 (63%) of them were admitted from home or health facilities located beyond 50 km from the hospital (SFDDH).

**Table 2:** Specific case fatality rates among patients with severe maternal morbidities at SFDDH, 2008 - 2010

Severe morbidity/mortality	Total morbidities (judged as primary causes)	Number of maternal deaths	Specific case fatality rate (%)
Abruptio placenta	19	3	16
Placenta praevia	16	0	0
Postpartum haemorrhage	67	2	3
Eclampsia	101	9	9
Severe obstructed labour	37	0	0
Ruptured uterus	38	4	11
Complications of abortion	25	6	24
Severe anaemia in pregnancy	30	4	13
Puerperal sepsis	15	2	13
Other severe morbidities	15	6*	40
Total	363	36	10

Note: *Other causes of maternal deaths were complications of HIV/AIDS in pregnancy (3), ruptured ectopic pregnancy (1), amniotic fluid embolism (1) and cardiac arrest during surgery (1).

Of all severe morbidities 149 (41%) occurred during hospital stay at SFDDH and this included 42% (16) of ruptured uterus, 48% (18) of severe obstructed labour and 63% (42) of severe postpartum haemorrhage. Development of severe complications before arrival at the hospital increased the risk of maternal death by almost three times (RR 2.9; 95% CI: 1.3 - 6.3) (Table 3). Although, not statistically significant, the use of first level health facilities after onset of obstetric complication before going to the first referral hospital reduced chances of death by 16% (RR 0.84; 95% CI: 0.44 - 1.63). The review indicated that 15% (8) of mothers living in villages located between 51 - 100 km who developed complications before arrival to SFDDH bypassed the nearby first level health facilities (dispensaries and health centres) which could have provided first line management and probably refer them for definitive management. Of the total patients with severe maternal morbidities 329 (91%) had only one severe morbidity, 29 (8%) had two and 5 (1%) had three severe morbidities.

**Table 3:** The association between maternal deaths and health care seeking behaviour among patients with severe morbidities at SFDDH.

Factors (exposure/control)	Total severe maternal morbidities^†^ n = 363	Maternal deaths n = 36	Risk Ratio (95% CI)
Places where the morbidity developed			
Before arrival at SFDDH	214 (59%)	29	
During hospital stay	149 (41%)	7	2.9 (1.3 - 6.3)
Where the patients sought care in the first place after onset of complication(s)			
Traditional birth attendants	5 (1%)	1	-
Dispensary/Health centre	137 (38%)	12	
Hospital (SFDDH)*	221 (61%)	23	0.8 (0.4 - 1.6)

**†**Severe morbidities include maternal deaths

### Means of transport for patients with obstetric complications

The majority of patients whose complications occurred before arrival at SFDDH used hired taxi/car (32%), ambulance (27%) and public transport (24%). Others used bicycle (11%), motorcycle (2%), and walking on foot (3%). The mean time spent waiting for transport to SFDDH from either home or the referring health facility was 83 minutes, the mean duration of transport was 128 minutes and the duration could be as long as 540 minutes i.e. almost 9 hours.

### Areas of substandard care

Of all maternal mortalities and severe morbidities 341 (94%) had at least one area of substandard care. Patient's related substandard care was identified in as many as 180 (50%) patients with severe morbidities. The most common patient's related substandard care was delay to seek treatment identified in 111 (31%) patients (Table 4). Health workers' related substandard care were found in 221 (61%) mothers with severe morbidities at SFDDH and 94 (69%) of those using the first level health facilities in the first place after onset of complications (Table 5). The most common health worker's related substandard care were delayed referral identified in 33 (24%), and delayed treatment within the facility found in 104 (29%) patients. Proportionally, delayed treatment within the facility (p < 0.001) and inadequate treatment or monitoring of labour (p < 0.05) were statistically significantly higher at SFDDH than in the first level health facilities. There was no MgSO4 in all dispensaries and health centres. Patients with eclampsia referred from these first level health facilities were given diazepam bolus to control fits.

**Table 4:** Patient and administration related substandard care for maternal mortalities and severe morbidities at SFDDH, 2008 - 2010

Substandard Care	Proportions n = 363
**Patient-related factors**	
Presence of at least one substandard care	180 (50%)
Never/Poorly attended ANC	52 (14%)
Delayed to seek treatment	111 (31%)
Intoxication by local herbs	3 (1%)
Others	31 (9%)
**Administration related substandard care**	
Presence of at least one area of substandard care	219 (60%)
Absence of essential drugs, supplies and equipment	22 (6%)
Absence of/inadequate blood for transfusion	10 (3%)
Long distance from where the complications started to SFDDH (>50 km)	32 (9%)
Poor ANC, but difficult to judge whether care provider's or administration related factors.	52 (14%)
Lack of ambulance	24 (7%)
The facility had ambulance but was not readily available	21 (6%)
Others	12 (3%)

Note: ANC = antenatal clinic care

**Table 5:** Health worker-related substandard care for maternal mortalities and severe morbidities at the health facility level

Areas of substandard care	First level HF* n = 137	SFDDH n = 363	Chi-squared test (P value)
Presence of at least one area of substandard care	94 (69%)	221 (61%)	2.6 (0.11)
Delayed treatment within the facility	15 (11%)	104 (29%)	17.2 (0.00)
Delayed referral to hospital with CEmOC services	33 (24%)	NA	NA
Referred while not on appropriate treatment	27 (20%)	NA	NA
Inadequate treatment or monitoring of labour	17 (12%)	77 (21%)	5.0 (0.03)
Wrong diagnosis	6 (4%)	22 (6%)	0.66‡
Wrong treatment with a correct diagnosis	7 (5%)	36 (10%)	0.11‡
Others	1 (1%)	26 (7%)	0.00‡

Note: *First level HF refers to dispensaries and health centres; HF = health facility; NA = Not applicable; ‡ Used Fisher's exact test.

More than half (59% i.e. 214) of severe maternal morbidities occurred before arrival at SFDDH and 44% of these were judged to be contributed by transport problems. It was also noted that even when ambulances were available sometimes women preferred to use public transport or hired vehicles because these were considered to be cheaper.

### Successes of the audit

A list of strategic interventions for quality care improvement were proposed and implemented as a result of audit. These included improvement of responsiveness to obstetric emergencies, workforce development, referral system improvement and advocacy on upgrading of the most remote health centres to provide CEmOC. A policy was developed to ensure readily availability of staff by identifying rooms within the hospital where doctors spend their nights when they were on call. This recommendation was implemented in an attempt to ensure prompt intervention to reduce delay to provide care within the health facility.

In addition to feedback to staff involved in care, weekly education meetings were carried out with intention to update the knowledge and skills of care providers. The main emphasis was put on management of specific obstetric conditions repeatedly identified with substandard care during audit. The training followed the national guidelines for management of obstetric complications. In addition, the district authority posted more midwives to support services provision in the labour ward. In an attempt to improve the referral system the district authority started to provide fuel for all institutional ambulances for all women with obstetric complications referred from the dispensaries and health centres. Before that, mothers requiring referral had to pay for fuel for the ambulance as much as 150 USD. Considering the poverty of most people in the catchment area and the fact that they had poor birth preparedness and readiness for complications, the previous practice delayed patients to reach the first referral district hospital (SFDDH). Equally important the audit team provided feedback to referring health facilities whenever the referral norms were grossly not adhered to.

The audit team recommended to the district authority to upgrade the most remote health centres to provide CEmOC services. As a result two health centres (Mlimba and Kibaoni) were upgraded in Kilombero district. Mlimba health centre is located 150 km from SFDDH and the upgrading was financially supported jointly by the government and the World Lung Foundation. CEmOC services were launched at Mlimba in July 2010 and remarkable results on the performance have been documented elsewhere [16]. Upgrading of the most remote health centres to provide CEmOC services was intended to reduce phase two delays (delays to reach care).

### Barriers to successful audit

The main problems which were encountered during this period included shortage of staff complemented by high attrition rates of labour ward midwives and weak feedback. High attrition rate was linked to increased salaries in the government health sector. A remarkable number of these staff opted to join the government and were posted to other areas where the local government had also shortage of staff. Attrition of staff complemented the shortage of care providers and this necessitated hospital administration to regularly look for new staff. Most of these were new graduates, less experienced and less skilled contributing to increased substandard care. Acute shortage of skilled staff in the maternity block was complemented by internal transfer of nurse-midwives to other departments within the hospital. In an attempt to address staff attrition, the hospital management successfully convinced the government to include the staff in the government payroll and second them to this hospital. Other barriers included lack of funds for on job training in emergency obstetric care, inconsistent replacement of drugs and essential supplies as well as inadequate participation of hospital and district decision makers in the audit meetings. Despite such irresponsiveness, regular reminders were made for them to take part in the audit process.

## Discussion

This study indicates that audits for maternal mortality and severe morbidity can be implemented even in rural resource limited settings where the magnitude of maternal mortality is immense [1,4,7,12]. Like many other reports a wide range of benefits of audit in maternity care have been demonstrated here including improved patient care, knowledge and behavioural change in patient care and cost-effective use of resources [17-20]. Our experience indicates that audit has a great potential to bring change by continuous identification of adverse maternal outcome, underlying factors, implementation and evaluation of interventions for the purpose of improving care.

Audit identified a wide range of substandard care for severe maternal morbidities in rural Tanzania. Although it is impossible to state with certainty how many severe maternal morbidities might have been saved through focused courses of action, the presence of at least one category of substandard care in 94% of all severe morbidities suggests that the vast majority of these in resource limited countries are preventable if more investment for maternity care is made [4,5]. The presence of at least one health workers' related substandard care in 61% - 69% of patients with severe morbidities in these health facilities suggests inadequate knowledge, skills, attitude, morale and responsiveness to obstetric complications. On the other hand, the presence of at least one administration related substandard care in 60% of the cases suggests serious weakness of the health care systems, contributing to the burden of adverse maternal outcome in resource limited countries.

The fact that patients had to travel long distances (up to 200 km) from home villages to SFDDH complimented by poor transport infrastructure and unreadily availability of transport in case of referral, suggests inequitable geographical distribution and poor accessibility of CEmOC services in remote rural Tanzania where the majority (77%) of Tanzanians live [21]. Inequitable distribution of EmOC services has been also reported in other resource limited countries with sparsely populated sub-national geographical areas [15]. These findings suggest the need to map the geographical distribution of EmOC services in resource limited countries and draw attention to underserved areas. In such places where there is limited access to CEmOC services, resulting in a delay to treat life threatening childbirth complications, maternity waiting homes (MWH) may be an intervention to consider [22,23].

Failure of the hospital decision makers to implement audit recommendations is worrisome and could be associated with demoralization of staff and failure to improve the quality of care as reported in other places [17,24,25]. In places where establishment of maternal mortality audits has led to improved quality of obstetric care, the success has been particularly attributed to strong leadership and accountability of both health providers and key decision makers [7,11,25,26]. Our findings recommend a more responsive health system from the level of the ministry of health down to the grass root levels. The key actors in the health sector in Tanzania are proposed to translate the lessons learnt into actions and intensify efforts to replicate the practice (audit) even in public hospitals where such interventions are uncommonly implemented [24].

### Limitations of the study

Maternal deaths that occurred at home after women had left the hospital were not included. This could have led to underestimation of maternal deaths. However, this was thought to be low and could not have changed significantly the results. The impact of audit on maternal mortality and morbidity was not carried out because of lack of baseline data due to poor record keeping and the fact that there was no observation period before it was introduced. There was no quantitative or qualitative analysis to ascertain whether the substandard care identified in the beginning of audit did not repeat significantly in the latter half of the study period. The fact that substandard care was taken to be the reason for potentially avoidable severe morbidities, while the same type of substandard care could have been also found in cases without adverse outcome posed a great challenge to the verdict [27].

## Conclusions

The findings resulting from introduction of audit in this rural district hospital strongly indicate that audit can be implemented even in rural settings in resource limited countries. Evidences from this study suggest that the vast majority of maternal deaths and severe disabilities are preventable even where resources are limited. Audit is an important keystone for maternity quality care improvement. Its success, however, depends on the commitment of all stakeholders including care providers and relevant decision makers to implement audit recommendations. Introduction of such audits is proposed even in rural settings in resource limited countries in order to improve the quality of maternity care.

## Abbreviations

CEmOC: Comprehensive Emergency Obstetric Care; CI: confidence intervals; MWH: maternity waiting homes; RCH: reproductive and child health; SFDDH: Saint Francis Designated District Hospital.

## Competing interests

The authors declare that they have no competing interests.

## Authors' contributions

ASN participated in design and took part in the audit process, wrote the manuscript. ABJ took part in the audit process and analysis and contributed to writing of the manuscript. DPU contributed to writing of the manuscript, JvR participated in design of the audit process and contributed to writing of the manuscript. All authors read and approved the final manuscript.

## Pre-publication history

The pre-publication history for this paper can be accessed here:

http://www.biomedcentral.com/1471-2393/11/94/prepub
